# Annual Meetings of Hospitals

**Published:** 1920-04-10

**Authors:** 


### ANNUAL MEETINGS OF HOSPITALS.

### Cancer Hospital.

At the annual Court of Governors of the Cancer Hospital, Fulham, held last week, it was reported that the diminution in the number of out-patients was due to the decrease in the number of military patients in the electrical department. Despite the fact that some of the wards were closed for a considerable period in 1919 for repairs, in-patients were only five less in number than a year previously, when they totalled 636. The daily average number of beds occupied during the period under review was eighty-two, and the total number of operations performed 444. The greater part of the radiography done at the hospital is for the detection of disease. There was a marked increase in this work last year, the number of patients examined being 2,966, as compared with 2,150 in 1918. The Chairman of the Medical Committee states that with regard to cancer and its treatment in the future, great help may, it is hoped, be expected from the Ministry of Health. The report of the Ministry of National Service, issued recently, on the physical examination of recruits for the Army, states that the physical standard of the manhood in various parts of the country differs greatly. Having these data to go upon, says the Chairman, there is reason for inquiring what are the conditions present to account for the variation of the physique, and the Ministry of Health ought to be able to say whether there is reason to suspect the existence of cancer districts and cancer houses. The accounts of the hospital for last year show an income of £14,935, and an expenditure of £29,387. The upkeep of cancer beds is 50 per cent. greater than those in a general hospital.

### Poplar Hospital for Accidents.

In view of the enormously increased prices, says the report of the Poplar Hospital for Accidents, adopted at the annual meeting of Governors last week, the rebuilding of the west wing of the institution, including the enlargement of the casualty and *x*-ray departments and the building of additional rooms for nurses and another children's ward, for which an appeal for funds has been made, has been postponed. Definite plans and approximate estimates are, however, to be obtained, and considerable repairs in repainting and renewing the whole of the outside of the hospital carried out without delay. The total income last year of £28,761 is the largest ever received, and will enable £10,000 to be put to the suspense account for rebuilding, making the total amount set aside for that purpose £29,079. Expenditure has increased, but fortunately not to the extent of the income. In-patients in 1919 numbered 1,871, and showed an increase of 125 as compared with the preceding year. Casualties and out-patients to the number of 43,383 were also dealt with during the past twelve months, while 103 patients benefited by treatment at the hospital's convalescent home at Walton-on-the-Naze. New dental cases of L.C.C. school children numbered 1,462, and their attendances amounted to 2,109. There were over 6,000 attendances for massage.

### Royal National Orthopædic Hospital.

At the annual meeting of Governors of the Royal National Orthopædic Hospital, presided over last week by Mr. E. Dean, it was reported that 1,121 in-patients, 422 civil, and 699 military, had been cared for in 1919, during which 190 beds were available. Each in-patient was resident on an average fifty days. New out-patients to the number of 4,278, as compared with 3,175 in 1918, were also treated. Expenditure during the period under review amounted to £26,390, while income was only £24,759, there being an excess of the former over the latter of £1,633. The proposed extension of the hospital, the plans of which have been approved by the King's Fund, will cost at least £130,000. Completed early last year, the boot and instrument making workshops connected with the institution are in active operation. Patients' payments during 1919, including £13,226 received from the War Office for soldiers, amounted to £14,214.

### Royal Westminster Ophthalmic Hospital.

The annual meeting of Governors of the Royal Westminster and Ophthalmic Hospital was held last week, when it was reported that, as after the Napoleonic Wars (the institution having been founded in 1816), the hospital continues to treat numbers of discharged soldiers as out-patients who are suffering from diseases of and injuries to the eye. An arrangement has been made with the Ministry of Pensions to open an evening clinic for the benefit of ex-Service men and pensioners. Income last year was only £4,339, having decreased by £2,143 as compared with 1918. Expenditure, on the other hand, had increased from £4,905 in that year to £5,648 during the following twelve months, expenditure thus exceeding income by £1,308.

### Royal Dental Hospital.

The immediate pressing want is more room for specialisation, pathology, and research work and the provision of a certain number of beds for patients who are undergoing serious facial treatment, as well as for cases which involve prolonged operations. Those persons, it is pointed out, are unfit to travel home, and require careful nursing to ensure a good recovery. The number of properly qualified dental practitioners, it is stated, is inadequate to the needs of the nation, there being at the present time in this country only 4,500 duly qualified men on the dental register. The dental school attached to the hospital is full to overflowing, and needs to be enlarged and improved. Income last year totalled £6,090, and exceeded the expenditure of £5,876 by £214. New subscribers, however, are earnestly appealed for.

### University College Hospital.

Sir Ernest Hatch presided last week over the annual Court of Governors of University College Hospital, which at the end of 1919 was faced with a deficit of £11,727, income having declined from £58,203 for 1918 to £43,411 during the twelve months following. Expenditure amounted to £57,482, as compared with £56,031 in 1918. The falling off in income is accounted for by the closing of the military wards. In-patients to the number of 4,253 were cared for last year, while 63,661 out-patients, whose attendances numbered 183,978, also received attention. The number of wounded soldiers treated as in-patients in 1919 was 177, bringing the total number accommodated since the outbreak of war up to 6,032. Over 1,160 pensioners, of whom 165 were in-patients, were dealt with last year under agreement with the Ministry of Pensions. A memorial tablet to the late Lord Lister, presented by the Lister Memorial Fund to the hospital, has been placed in a prominent position at the head of the staircase leading from the main entrance corridor. The tablet, which is of white marble, embodies a medallion portrait in relief, and an inscription recording the fact that Lord Lister was a student and house surgeon at the hospital from 1843 to 1852.

### Metropolitan Hospital.

Lord Howard de Walden presided over the annual meeting of Governors of the Metropolitan Hospital, held last week. A special sub-committee has been appointed to consider the expenditure of the hospital under its various headings. Last year expenditure exceeded income by over £4,100. The committee has decided, after careful consideration, to make an experiment in the admission of paying patients, and one ward will be opened very shortly for their reception. It is not, however, in favour at the moment of making a regular charge to every patient of a fixed sum for their food. The suggestion has been considered, but rejected. The institution, it is recalled, was originally named the Metropolitan Free Hospital, and patients have never hitherto been required to provide anything for themselves. It is believed that the people resident in the district surrounding the hospital, who have always regarded it as their own, will rally to its support, and that a strong appeal, signed by the Lord Mayor, the Lady Mayoress, and the local Mayors, which is about to be issued, will not be made to them in vain.

